# The burden of thyroid eye disease: Evaluating treatment outcomes on quality of life

**DOI:** 10.1177/11206721261422570

**Published:** 2026-02-11

**Authors:** Abbie C. Lai, Brian E. Yu, Monali Malvankar-Mehta

**Affiliations:** 1 Michael G. DeGroote School of Medicine, 12362 McMaster University, Hamilton, Ontario, Canada; 2 Schulich School of Medicine and Dentistry, 70384 Western University, London, Ontario, Canada; 3 Department of Ophthalmology, Western University, London, Ontario, Canada; 4 Department of Epidemiology and Biostatistics, Western University, London, Ontario, Canada

**Keywords:** Thyroid eye disease, graves’ orbitopathy, graves’ ophthalmopathy, quality-of-life, mental health

## Abstract

**Background:**

Thyroid eye disease (TED) is characterized by inflammation of orbital tissue, proptosis, diplopia, and potential changes in facial appearance. TED has been associated with poor mental health and reduced health-related quality of life (HRQOL) due to the combined impact of impaired visual functioning and changes in facial appearance. The study aims to determine the impact of treatments for TED on patients’ HRQOL as a measure of comprehensive well-being.

**Methods:**

Systematic searches of literature in MEDLINE, EMBASE and CINAHL databases and grey literature sources were conducted. After de-duplication and screening, the modified Downs-and-Black criteria were used to assess the risk of bias. Baseline characteristics, HRQOL-questionnaire data, and mean HRQOL measurements were extracted.

**Results:**

Searches yielded 313 studies from databases and 9 grey literature studies. 23 studies (2023 patients total) were included for quality assessment and data synthesis. Strabismus surgery yielded the greatest improvement in total HRQOL score. Surgical interventions improved visual function QOL (VF-QOL) and visual appearance QOL scores (Ap-QOL).

Other medical therapies, including doxycline and selenium were effective for improving Ap-QOL.

**Conclusion:**

Surgical interventions, particularly decompression, eyelid, and strabismus surgery, improved appearance-related QOL. Immunosuppressive therapy and doxyxycline contributed to progressive functional improvements over time. Longer follow-up durations were generally associated with greater patient-reported benefit from treatments.

## Introduction

Thyroid eye disease (TED) is an autoimmune condition associated Graves’ disease, affecting 25% of patients with Graves’ hyperthyroidism and with varying regional prevalence ranging from 27% in North America to 58% in Oceania.^1^ It can also develop in euthyroid or hypothyroid individuals and in association with other autoimmune thyroid disorders such as Hashimoto thyroiditis.^2^

Psychological care is notably underemphasized in Graves’ disease (GD) care, despite the association of disease with increased suicide risk.^3^ TED has also been associated with loneliness, depression and anxiety, with the combined impact of impaired visual functioning and changes in facial appearance contributing significantly to reduced health-related quality of life.^4^ This may be due to a variety of reasons including impaired vision and facial disfigurement.

Characterized by inflammation of the orbital tissues, TED leads to a range of visual and cosmetic symptoms, including eyelid retraction, proptosis and restrictive extraocular myopathy. These symptoms may be associated with visual disturbances such as blurry or double vision, lacrimation, photophobia and even optic nerve compression, all of which may impair vision.^5^ Many individuals may struggle with daily tasks like driving. Patient-reporting tools like the Graves’ Ophthalmology Quality of Life Questionnaire (GO-QOL) are designed with patients’ experiences of the disease and symptoms management in mind. The GO-QOL consists of two sub-sections (visual functioning (VF) and visual appearance (VA)), where a higher score indicates better health-related QOL (HRQOL).^6^ These scores may be used to measure patients’ challenges with managing symptoms of impaired vision and aesthetic disfigurement.

Treatment for TED includes both medical (e.g glucocorticoid, small-molecule, selenium) and surgical (e.g orbital decompression) interventions depending on disease phase and severity. Medical treatments like immunosuppressive therapy with corticosteroids help with reducing swelling while orbital decompression surgery is the preferred intervention for cases involving optic nerve compression or severe proptosis.^2^ The treatment of TED should be tailored to each patient, emphasizing a holistic, patient-centered approach that addresses both its physical and psychological effects.

While the association between TED and mental health conditions is known, the comparative effects of different treatments on patient's quality of life is not known. The aim of this systematic review is to evaluate how different treatment modalities affect patients’ perceived quality of life. HRQOL results will be used as a comprehensive measure of mental and physical well-being. This review will address the impact of treatments for TED on patient's perceived personal wellness, encompassing functional status, confidence and self-esteem.

## Methods

### Search design

**Figure 1.**
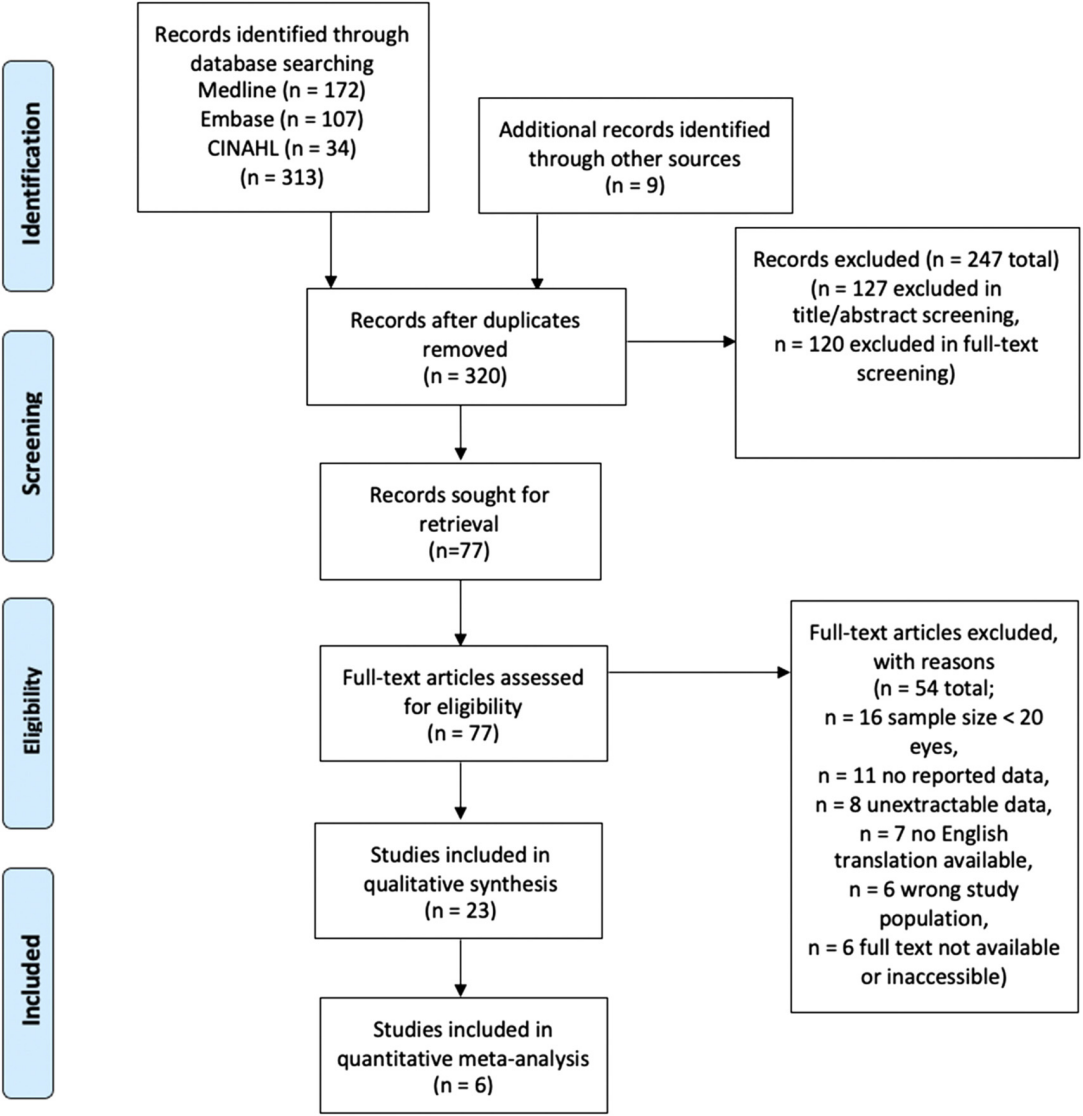
PRISMA flowchart depicting results from the literature search.

In this systematic review, Preferred Reporting Items for Systematic Review and Meta-Analyses (PRISMA) guidelines were followed.^7^ Figure 1 displays the detailed PRISMA flowchart. Searches were carried out over MEDLINE/PubMed (Ovid), EMBASE (Ovid), and CINAHL (EBSCO) databases. Grey literature was also searched, including ProQuest Dissertations and Theses Global, and conference abstracts held through the American Academy of Ophthalmology (AAO) and the Association for Research in Vision and Ophthalmology (ARVO). Search strategies designed for each source comprised the keywords presented in Appendix A. Published and unpublished studies were searched between July 2018 until May 202

### Screening

Randomized control trials, clinical trials, multicenter studies, comparative studies and observational studies were included for meta-analysis. Studies in the English language and conducted on at least 20 human orbits in subjects aged 18 or older with TED were included. Studies measuring HRQOL, anxiety, depression, psychological distress or other measures of psychological health prior to and following intervention were included. Studies were excluded if they had no data reported or were not available in English text or translation.

Results were imported into Covidence (Systematic review software, Veritas Health Innovation, Melbourne). After removing duplicates, two levels of screening were conducted by two independent reviewers (A.L. and B.Y.) Appendix B provides details on screening questions. At each stage, agreements and disagreements were merged and conflicts were resolved. Cohen's kappa coefficient (Appendix C) was computed at each level of screening.

### Quality assessment

A modified Downs and Black checklist was used to assess the methodological quality of studies, including risk of bias (Appendix D).^8^ The checklist includes questions to critically appraise study methodologies by evaluating external validity, study bias, confounding and selection bias, and the power of the study.

### Data extraction

The following information was extracted from studies with a quality score of at least 17 (out of 27): author(s), year of publication, country of origin, study design, sample size, treatment and control groups, size of each group, mean age of participants in each group, percentage of female participants in each group, testing period, follow up period, baseline clinical activity scores (CAS) and QOL questionnaire type. Various HRQOL assessments were reported including disease specific questionnaires (Graves’ ophthalmopathy QOL, TED-QOL, ThyPRO), generic health surveys (SF-36, SF-12). Treatments included radiotherapy, hormones, antibodies, antibiotics, enzyme inhibitors, minerals, and surgical procedures.

### Data-analysis

Due to the clinical heterogeneity of TED phenotypes and the variability in intervention targets, we performed a narrative synthesis stratified by treatment, using pre- and post-treatment HRQOL scores. In many clinical settings, patients have mixed phenotypes and receive treatments with different targets (e.g decompression surgery for proptosis and optic neuropathy).

This approach provides more nuanced clinical distinctions by reflecting real-world clinical decision-making for TED. Studies assessing patient-reported outcomes using instruments not directly comparable with the GO-QOL were retained for narrative contextualization.^9,10^

## Results

Database searches yielded 313 studies including 172 from MEDLINE, 107 from EMBASE and 34 from CINAHL. 9 additional texts were found from grey literature sources. After screening, 77 studies remained for full-text extraction. The KAPPA statistics for agreement between two reviewers were 0.38 and 0.57 for title and abstract screening. Following full-text screening and quality assessment, data was extracted from 23 studies that took place in 13 countries, Bulgaria, Brazil, Canada, China, Germany, Iran, Israel, Italy, Netherlands, Taiwan, Thailand, United Kingdom, USA. The treatments assessed in these studies were intravenous glucocorticoids (IVGC), antibiotics, decompression surgery, eyelid surgery, fat resection surgery, immunosuppressive therapy, IV glucocorticoids, minerals (selenium), orbital rim removal surgery, pentoxifylline, placebo, radiotherapy, small molecule treatment (doxycycline), squint surgery, strabismus surgery, teprotumumab and thyroidectomy. Characteristics of the studies are summarized in Table 1. Reported adverse events and complications from the included studies are summarized in Table 2.

**Table 1.** Study characteristics of articles included in data extraction.

Author (Year of publication)	Study design	Study location	Participant groups	N	Mean age (SD)	%Female participants
Almeida et al. (2024)	Randomized controlled trial (RCT)	Brazil	Inferomedial wall orbital decompression Balanced medial plus lateral wall orbital decompression	42	47.5 (12.7) 49.9 (10.9)	67
Bartalena et al. (2012)	Randomized controlled trial (RCT)	Europe (8 EUGOGO centres)	IVGC low (2.25 g over 12 wks)	53 54 52	54 (10)	70
Campi et al. (2021)	Cross-sectional	Italy	GD patients, healthy controls	100	53 (13.5)	74
Cheng et al. (2018)	Retrospective chart review	Taiwan	IVGC medium (4.98 g over 12 wks)	53	50 (8)	74
Douglas et al. (2020)	RCT	USA, Europe	IVGC high (7.47 g over 12 wks)	41 42	56 (11)	81
Fayers et al. (2016)	Prospective	Canada	Decompression surgery Strabismus surgery Eyelid surgery	29 15 21	NR	79
Fichter et al. (2013)	Descriptive	Germany	Decompression surgery, orbital rim removal surgery, fat resection surgery	18	48.1	72
Hoppe et al. (2020)	Prospective follow-up	Germany	GO patients	100	50.6 (10.7)	72
Jellema et al. (2014) *	Prospective	Netherlands	Strabismus	28	54.5 (11.2)	71
Jellema et al. (2017) *	Prospective	Netherlands	Strabismus surgery	59	57 (11.5)	66
Kahaly et al. (2005)	Prospective	Germany	Oral steroid treatment IVGC	70	48 52	70
Kashkouli et al. (2011)	Prospective, Cross-sectional	Iran	Steroid treatment, decompression surgery	61	38.3 (13.4)	64
Lane L. C. et al. (2021)	Cross-sectional	United Kingdom	GD patients, healthy controls	26	18	81
Liang et al. (2024)	Cohort observational study	China	Dysthyroid optic neuropathy Non-dysthyroid optic neuropathy	194	52.42 (9.64) 50.86 (8.47)	59
Lin et al. (2015)	Cross-sectional	China	Doxycycline	13	43.38 (11.82)	23
Marcocci et al. (2011)	RCT (Randomized, double-blind, placebo-controlled)	Netherlands	Selenium Pentoxifylline Placebo	152	48 37 41	83
Mourits et al. (EUGOGO) (2009)	Prospective	Europe (8 EUGOGO centres)	Orbital decompression	139	47.6	74
Potita et al. (2024)	RCT	Thailand	Selenium Placebo	25	42.4 (12.7) 48.1 (1.6)	72
Prummel et al. (2004)	RCT (Double-blind)	Netherlands	Radiotherapy, placebo	88	45.15	81
Smith et al. (2017)	RCT	USA	Teprotumumab Placebo	43 44	51.6 (1.06) 54.2 (13)	65 82
Stoynova et al. (2024)	Cross-sectional	Bulgaria	Mild to severe GD	171	52 (11.6)	77
Terwee et al. (2001)	Prospective	Netherlands	Radiotherapy Decompression surgery Eye muscle surgery Eyelid surgery Blepharoplasty surgery	164	57 (10) 56 (13) 43 (19) 56 (13) 50 (12)	75 100 80 68 86
Zloto et al.	Retrospective chart review	Israel	IVGC (EUGOGO recommendations), immunosuppressive treatment, decompression surgery, strabismus surgery, eyelid surgery	132	61.04 (16.88)	71.21

*Note: indicated studies are distinct studies involving different interventions from different years.

**Table 2.** Reported rates of adverse events and complications.

Author	N	Questionnaire	Participant groups	Adverse reaction	Adverse rate (%)
Douglas et al.	41	GO-QOL	Teprotumumab	Muscle spasm	32
Douglas et al.	42	GO-QOL	Placebo	Muscle spasm	10
Douglas et al.	41	GO-QOL	Teprotumumab	Alopecia	20
Douglas et al.	42	GO-QOL	Placebo	Alopecia	12
Douglas et al.	41	GO-QOL	Teprotumumab	Dry skin, dysgeusia	10
Fichter et al.	18	GO-QOL	Decompression	Skin retraction	21.73913043
Kahaly et al.	102	SF-36	No treatment	Weight gain	26
Kahaly et al.	102	SF-36	No treatment	GI issues	17
Kahaly et al.	102	SF-36	No treatment	Sleeplessness	14
Kahaly et al.	102	SF-36	No treatment	Palpitations	11.42857143
Mourits et al.	139	GO-QOL	Decompression (3 wall coronal procedure)	Dysesthesia	35.71428571
Mourits et al.	139	GO-QOL	Decompression (3 wall coronal procedure)	Eyelid swelling	21.42857143
Mourits et al.	139	GO-QOL	Decompression (3 wall coronal procedure)	Paralysis frontal muscle	21.42857143
Mourits et al.	139	GO-QOL	Decompression (2 wall swinging eyelid procedure)	Eyelid swelling	28.57142857
Potita et al.	25	GO-QOL	Selenium	Cutaneous irritation	8
Potita et al.	25	GO-QOL	Selenium	GI issues	4
Prummel et al.	88	GO-QOL	No treatment	Myalgias/arthralgias	16.66666667
Prummel et al.	88	GO-QOL	Rituximab	Myalgias/arthralgias	15.38461538
Prummel et al.	88	GO-QOL	Rituximab	Optic neuropathy	7.692307692
Prummel et al.	88	GO-QOL	Rituximab	Skin irritation (rash, itching)	15.38461538
Prummel et al.	88	GO-QOL	Rituximab	Lacrimation	7.692307692
Smith et al.	87	GO-QOL	Teprotumumab	Nausea	19
Smith et al.	87	GO-QOL	Teprotumumab	Muscle spasm	19

### Summary of go-QOL scores

#### Baseline scores

Stoynova et al. found patients with mild TED had higher VF QOL scores (82.0 ± 15.3, p < 0.01) compared to moderate-to-severe (54.6 ± 27.2, p < 0.01) and sight-threatening GO (27.3 ± 20.0, p < 0.01) at baseline. Furthermore, higher CAS, diplopia score, worse visual acuity, and greater symptom severity were associated with lower visual function scores (R^2^ = 0.44, p < 0.01). Female gender, higher CAS, diplopia score, and greater proptosis were associated with lower appearance scores (R^2^ = 0.39, p < 0.01).^11^

In a cross-sectional study, dysthyroid optic neuropathy (DON) patients had significantly lower visual function scores (33.18 vs. 81.26) compared to non-DON patients.^12^

#### Surgical treatments

Surgical interventions, including decompression, strabismus, and eyelid surgeries, demonstrated varying degrees of improvement in visual function and visual appearance for patients with TED. Cheng et al. and Fichter et al conducted retrospective studies, both demonstrating improved VF scores with decompression surgeries (p < 0.01, p = 0.016 respectively; Table E1).^13,14^ Almeida et al. (2024) compared inferomedial wall orbital decompression and medial plus lateral wall orbital decompression, which both significantly reduced exophthalmos by 2.4 and 3.8 mm respectively (p < 0.001). Only medial plus lateral decompression significantly improved appearance scores (p = 0.006). There was no significant change in visual functioning scores (p = 0.362, p = 0.727).^15^

The prospective study by Jellema et al. reported an increase in VF scores (p = 0.009) and VA scores (p = 0.005) in response to strabismus surgery (Tables E1 and E2).^16^

Fayers et al. reported improved visual appearance with all procedures (decompression, strabismus, and eyelid surgeries; p = 0.038, p = 0.016, and p = 0.0003, respectively; Table E2). VF score improved with strabismus surgery (p = 0.0048) and eyelid surgery for palpebral aperture narrowing (p = 0.1244). However, VF was scored worse with decompression surgery (p = 0.94).^17^

#### Steroid treatments

Two studies investigated the effects of methylprednisolone on GO-QOL. Hoppe et al. found improved visual functioning at 6 months and 9 months (p = 0.01, p = 0.04 respectively) and significant improvement in scores over time (Tables E1, E2 and E3). Furthermore, visual appearance also improved at all follow ups (p = 0.01, 0.005).^18^ Bartalena et al. found dose-dependent increase in VF scores with treatment over 12 weeks (p < 0.05, Table E1).^19^

#### Combined steroid and surgical treatments

Multiple studies compared the impact of various treatments within the same study population. Campi et al. reported significant improvements in visual function and visual appearance in patients responding to IVGC treatment (p < 0.05) and squint surgery (p < 0.01). In this study, patients were considered “responsive” to treatment based on a post-treatment Clinical Activity Score (CAS) < 3. The effect of steroid treatment on HRQOL was associated with response to treatment, while squint surgery was associated with improved VF scores despite response to treatment (Tables E1 and E2).^20^

Kashkuoli et al. found improved visual function with both glucocorticoid treatment and decompression surgery (p = 0.06; Table E1).^21^ Similarly, Zloto et al. found significantly higher overall GO-QOL scores (p < 0.001) in groups receiving IVGC, immunosuppressive therapy and surgical interventions compared to untreated controls (Table E3).^22^

#### Small-molecule treatments

Lin et al. performed a cross-sectional study demonstrating improved visual function and visual acuity with doxycycline antibiotic treatment (p = 0.21, p = 0.0008 respectively; Tables E1 and E2).^23^

#### Biologics

Smith et al. conducted a RCT showing improved visual function with teprotumumab antibody treatment compared to the placebo group (p < 0.001; Table E1).^24^

Marcocci et al. found improved visual function and visual appearance with selenium antioxidant treatment compared to placebo (p < 0.05, p < 0.001 respectively; Tables E1 and E2).^25^ Conversely, Potita et al. found no significant change in GO-QOL scores in the selenium group, but did note reduction in palpebral aperture (−1.4 ± 1.7 mm, p = 0.04).^26^

#### Radiotherapy treatment

In a RCT, Prummel et al. assessed the impact of radiotherapy on GO-QOL. Counterintuitively, the placebo group exhibited higher VF scores than the radiotherapy group (Table E1). However, the radiotherapy group demonstrated slightly improved VA compared to the placebo group (Table E2).^27^

#### Radiotherapy compared to surgical treatment

Terwee et al. conducted a prospective study evaluating the impact of various treatments on GO-QOL. Both eye muscle surgery and blepharoplasty improved visual function scores, with blepharoplasty also enhancing visual appearance. Decompression surgery led to improved overall GO-QOL. While radiotherapy showed improved VF scores, further research is needed to confirm these findings due to the relatively small effect size (p = 0.05).^28^

### Changes in QOL scores across treatments groups

**Figure 2.**
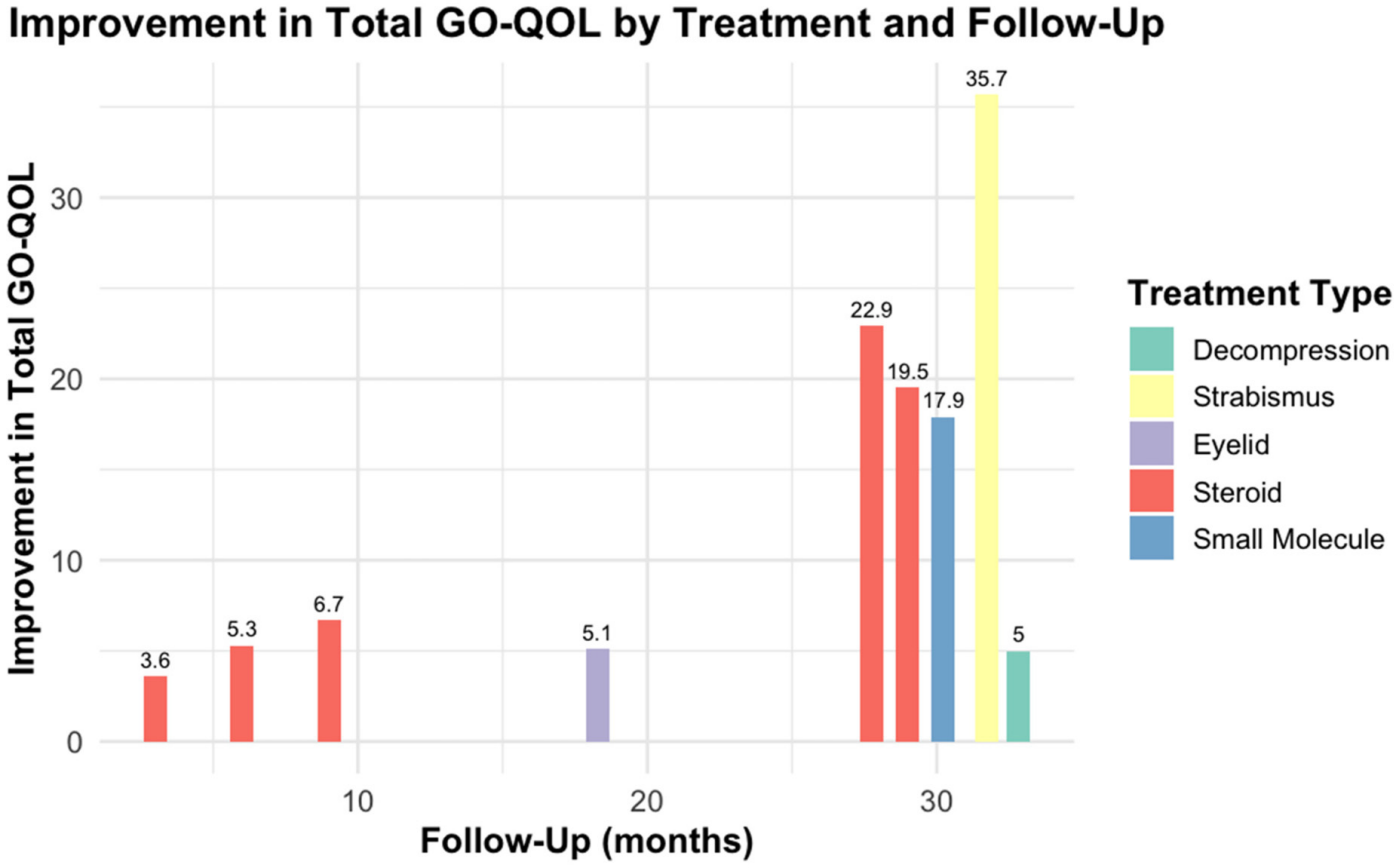
Improvement in total go-QOL score stratified by treatment groups at different follow-up durations. Each bar represents an individual study observation, color-coded by treatment category as seen in the corresponding legend.

Changes in total QOL score stratified across treatment groups can be seen in Figure 2, with improvement in scores with extended follow-up intervals, particularly with IVGC from 0 to 30 months. Strabismus surgery had the greatest improvement in GO-QOL at 30 months (Figure 2).

**Figure 3.**
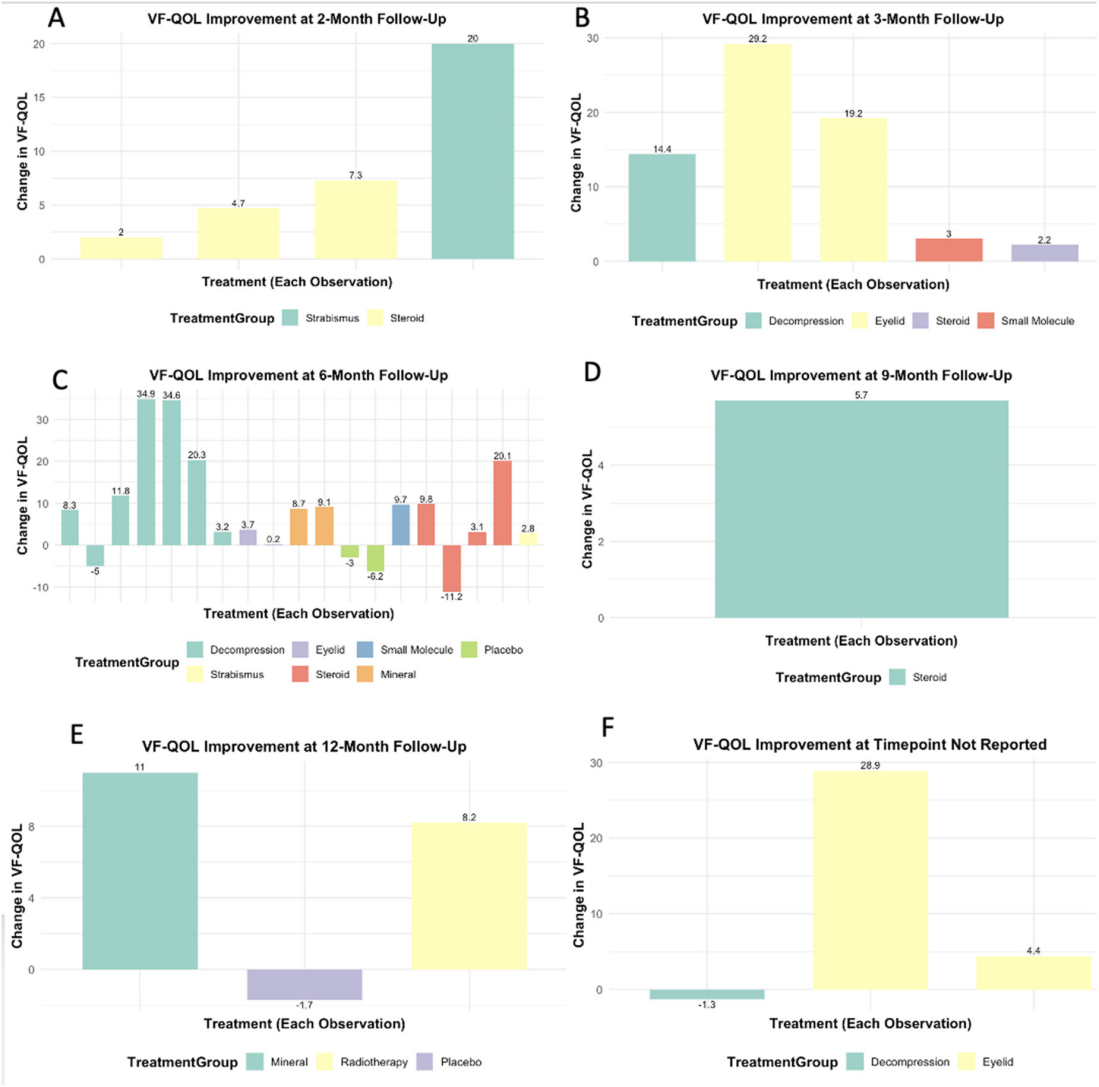
Improvement in visual function QOL (vf-QOL) by treatment groups at different follow-up durations.

Strabismus surgery notably improved visual function QOL scores early at 2 months. Eyelid surgery similarly substantially improved VF-QOL at 3 months and an unspecified timepoint. Decompression surgery had some mixed results at 6 months, but generally improved VF-QOL. Steroid treatment generally improved VF-QOL as time progressed up to 9 months of follow-up. Mineral supplement yielded increasing improvement on VF-QOL from 6 to 12 months. Overall, all treatment generally improved patient-perceived vision compared to placebo, which consistently worsened VF-QOL scores (Figure 3).

(A–F) Each panel displays the mean change in VF-QOL scores reported at timepoints:

(A) 2 months, (B) 3 months, (C) 6 months, (D) 9 months, (E) 12 months, and (F) timepoint not reported. Each bar represents an individual study observation, color-coded by treatment category as seen in each corresponding legend.

Panels (A–F) display the mean change in Ap-QOL score at the following timepoints:

(A) 2 months, (B) 3 months, (C) 6 months, (D) 9 months, (E) 12 months, and (F) timepoint not reported. Each bar represents an individual study observation, color-coded by treatment category as seen in each corresponding legend.

**Figure 4.**
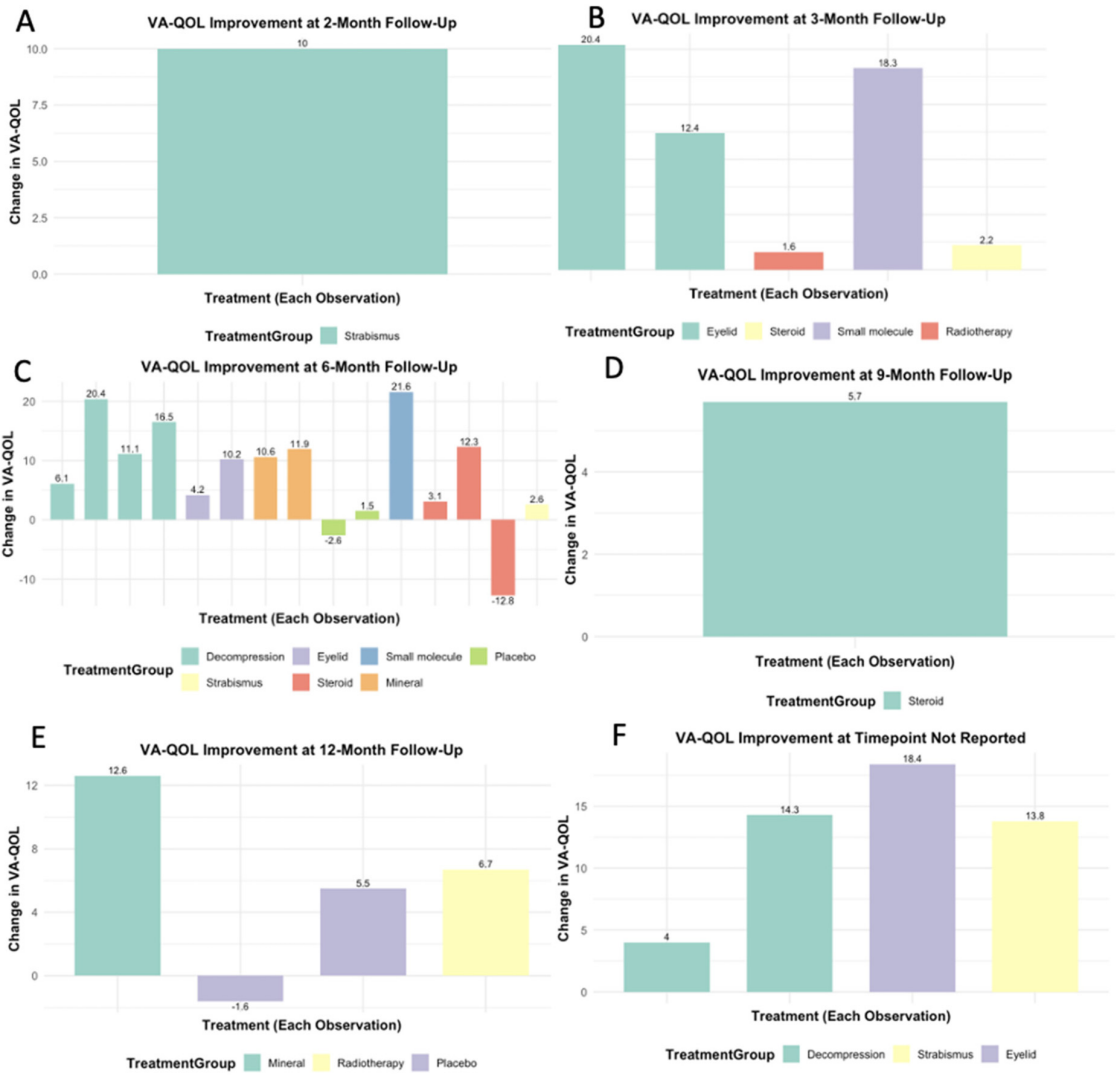
Improvement in visual appearance QOL (ap-QOL) by treatment groups at different follow-up durations.

Greatest improvements in Ap-QOL were seen with eyelid and decompression surgery at 3 and 6 months respectively, and at an unspecified timepoint. Strabismus surgery showed early improvement at 2 months and at an unspecified timepoint. Small molecule treatment with doxycycline also notably improved Ap-QOL at 3 and 6 months. There were some mixed results seen with steroids at 6 months, with improvement in Ap-QOL by 9 months. Placebo resulted in mixed Ap-QOL changes at 6 and 12 months. Mineral treatment with selenium improved Ap-QOL at 6 months and 12 months (Figure 4).

### Adverse effects of treatments

Musculoskeletal symptoms like muscle spasms, myalgia and arthralgia were common adverse effects among biologics like monoclonal antibody medications (teprotumumab and rituximab)^24,27,29^ (Table 2). Surgical procedures, like decompression surgery, were associated with neurological symptoms such as dysesthesia and frontal muscle paralysis, as well as general side effects like swelling. Skin symptoms such as alopecia, skin retraction, and irritation were present across medicinal and surgical treatment (Table 2).

## Discussion

The European Group on Graves’ Orbitopathy (EUGOGO) guidelines emphasize the importance of addressing both physical and mental health aspects of TED for comprehensive management.^2^ This systematic review investigated the impact of different classes of treatments for TED on patients’ perceptions of visual function and visual appearance. In alignment with EUGOGO recommendations, our findings highlight the importance of surveying patients’ perceptions of treatments on their lives to better understand psychological well-being and tailoring treatments based on patients’ goals. A holistic approach integrating both medical and psychological aspects may lead to improved patient satisfaction and treatment adherence.

There are several proposed mechanisms describing the impact of TED on psychological health. From the cross-talk between the endocrine and neuropsychiatric systems to the presence of antibodies targeting TSH receptors in neuronal tissue, multiple theories attempt to explain the association of mood and anxiety disorders in patients with TED and other endocrine disorders.^30–33^ These neuropsychiatric components of TED support the integration of mental health care in treatment plans. The interplay between TED severity and QOL highlights how each can both predict and exacerbate the other. The predictive model developed by Liang et al. uses GO-QOL scores to identify patients at risk for DON, suggesting integration of HRQOL scores into screening protocols may facilitate earlier intervention.^12^

While steroids are typically prescribed for active inflammatory disease process, decompression surgery for proptosis or optic neuropathy, and strabismus surgery for restrictive myopathy, patient presentations and indications for treatment were more heterogenous. Our treatment-stratified synthesis enables clinical insight while preserving study-level context to evaluated treatment impact on patient-reported outcomes. Total GO-QOL score improvements were seen across all treatment groups but especially with strabismus surgery. Surgical treatments, particularly decompression, eyelid and strabismus surgery, were associated with the greatest improvements in appearance-related Ap-QOL. Strabismus surgery contributed to early improvements at 2 months while eyelid and decompression surgeries were more impactful at 3 and 6 months. These findings align with prior research indicating that the psychosocial burden of TED is heavily influenced by disfigurement, reinforcing the value of surgical intervention in restoring patient confidence and mental well-being.^14,16,21^ Surgical interventions also improved visual function QOL, with strabismus leading the greatest score improvement, which may be associated with improved muscle control and reduced diplopia.^34^

Steroid treatments demonstrated progressive benefits in VF-QOL and Ap-QOL over time, particularly by 9 months. In the literature, the effects of methylprednisolone are similarly mixed.^19,35,36^ The reason may be traced to the role of methylprednisolone to primarily reduce orbital inflammation, which does not directly correlate with improving visual function.^18,19,35–37^ Given the delay in symptom relief, early involvement of a multidisciplinary team, including psychiatrists, neurologists, ophthalmologists, and endocrinologists, may provide crucial psychological support during this extended recovery phase.

Longer follow-up was generally associated with greater gains, especially with IVGC up to 30 months improving total GO-QOL. Similarly, mineral treatment yielded increasing improvement on VF-QOL over time. Radiotherapy treatment increasingly improved Ap-QOL from 3 to 12 months, and also resulted in modest improvement in VF-QOL. A period of 6 to 18 months has been generally recommended for recovery for all TED treatments (medical, surgical, or radioiodine).^38,39^ Our results indicate substantial patient perceived improvement in quality of life beyond this time frame.

Further work may investigate optimal surgical approaches and recovery timelines from patients’ perspectives. For instance, the differentiation between inferomedial and medial plus lateral wall decompression provides further granularity in surgical planning for functional and aesthetic outcomes.^15^ Other evidence suggests both lateral orbital wall decompression and balanced decompression techniques yield similar improvements in visual appearance, allowing for greater flexibility in surgical planning.^39,40^

The review of adverse effects associated with both pharmacological and surgical treatments for TED is presented alongside their therapeutic benefits. These findings underscore the importance of comprehensive patient counseling to manage expectations regarding potential musculoskeletal and neurological complications. With these findings, clinicians can better manage patient expectations and tailor treatment plans to provide care for the physical and psychological symptoms of this disease.

A limitation of this review is the inherent heterogeneity in TED phenotypes across included studies. Despite efforts to categorize clinical presentations (e.g., inflammatory, myopathic, proptosis/cosmetic, optic neuropathy), overlapping clinical features frequently necessitated multi-target interventions. Future research could refine subgroup analyses by employing standardized phenotype definitions and stratifying outcomes by distinct clinical presentations.

## Conclusion

Given the profound impact of TED on psychological and physical well-being, this study provides a comprehensive assessment of how different treatment modalities influence patients’ perception of their health. While treatment efficacy varied across group, most interventions, particularly steroid and mineral supplementation, improved patient perceived outcomes particularly with longer follow-up. Surgical treatments including eyelid, strabismus and decompression surgery, had substantial improvement on appearance-related QOL. These findings suggest that surgical intervention is particularly effective for patients who primary concern is disfigurement, given the correlation of visual appearance with psychological distress. Furthermore, predictive models using HRQOL scores to identify DON patients highlight the potential for using HRQOL scores to facilitate earlier intervention and better patient outcomes.

Future research may focus on refining patient selection criteria, standardizing phenotype classifications and developing combination therapies that enhance both vision and appearance outcomes. An individualized treatment strategy is recommended for all patients, incorporating pharmacological, surgical and psychological support. The management of TED requires a multidisciplinary approach to address the diverse factors impacting patient HRQOL. By adopting a multidisciplinary, evidence-based approach, clinicians can provide more effective, patient-centered care for individuals with TED.

## Supplemental Material

sj-docx-1-ejo-10.1177_11206721261422570 - Supplemental material for The burden of thyroid eye disease: Evaluating treatment outcomes on quality of life Supplemental material, sj-docx-1-ejo-10.1177_11206721261422570 for The burden of thyroid eye disease: Evaluating treatment outcomes on quality of life by Abbie C. Lai, Brian E. Yu and Monali Malvankar-Mehta in European Journal of Ophthalmology
