# Supplementary material for: The burden of thyroid eye disease: Evaluating treatment outcomes on quality of life
Source: Eur J Ophthalmol. 2026 Feb 11;36(4):1061–72. doi: 10.1177/11206721261422570 (PMC13269726; doi:10.1177/11206721261422570)
Supplement: sj-docx-1-ejo-10.1177_11206721261422570 - Supplemental material for The burden of thyroid eye disease: Evaluating treatment outcomes on quality of life [file sj-docx-1-ejo-10.1177_11206721261422570.docx]

**Supplementary Materials**

Appendix A. Search strategies

**Subject Headings & Term Harvesting**

| Concept | Medline | EMBASE | CINAHL | Keywords |
| --- | --- | --- | --- | --- |
| Graves’ eye disease | Graves Ophthalmopathy, graves orbitopathy, thyroid associated ophthalmopathies, dysthyroid ophtahlmopathies, graves’ eye disease, thyroid eye disease, congestive ophthalmopathy, infiltrative opohthalmopathies, edematous ophthalmopathy, myopathic ophthalmopathy | Endocrine ophthalmopathy, dysthyroid eye disease, dysthyroid ophthalmopathy, dysthyroid orbitopathy, endocrine orbitopathy, Graves eye disease, Graves ophthalmopathy, Graves orbitopathy, thyroid associated eye disease, thyroid associated ophthalmopathy, thyroid associated orbitopathy, thyroid eye disease, thyroid ophthalmopathy, thyroid orbitopathy, thyroid related orbitopathy | Graves’ Ophthalmopathy/ | Dysthyroid/ OR Graves eye disease OR Graves ophthalmopathy OR Graves orbitopathy |
| Psychological distress | Psychological distress, psychological stress, depressive disorder, anxiety, depression, psychosocial functioning | Distress syndrome, adolescent depression, depression, minor depression, major depression, mixed anxiety and depression, endogenous depression, organic depression, anxiety, generalized anxiety disorder, anxiety disorder, psychosocial disorder, psychosocial withdrawal |  | Anxiety disorder*/ OR mental disease |
| Quality of life | quality of life, psychological well being | Psychosocial adjustment to illness scale, quality of life, quality adjusted life year | Quality-adjusted life years/ OR Quality of Life (Iowa NOV)/ OR Health and Life Quality (Iowa NOV)/ OR Ferrans and Powers Quality of Life Index/ OR Physical Activity (Omaha) |  |

**Medline OVID**

Date: 12/13/2022 updated 05/15/2025


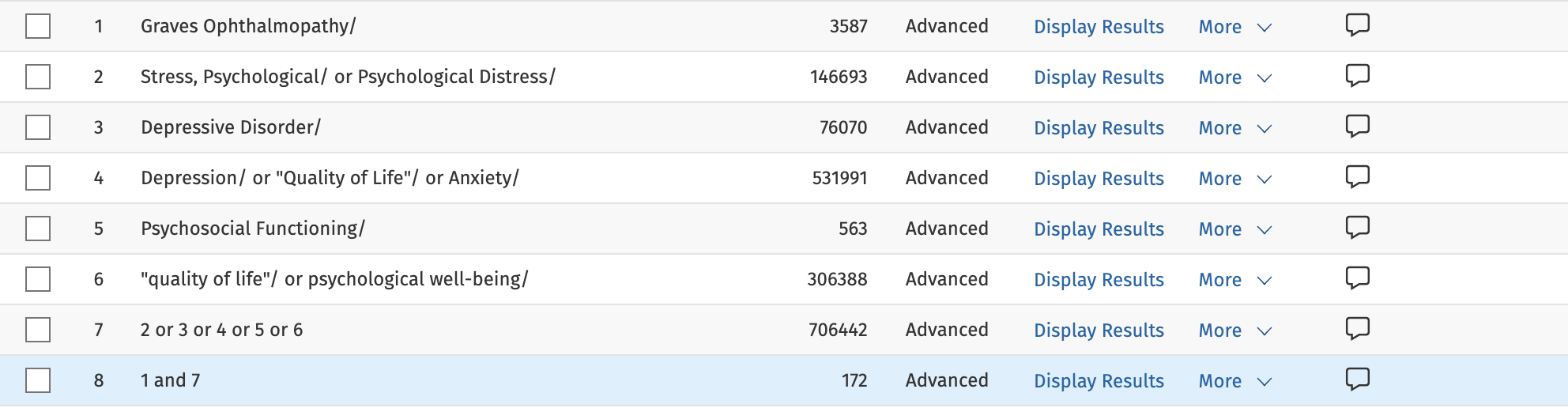


**EMBASE**

Date: 12/13/2022 updated 05/15/2025


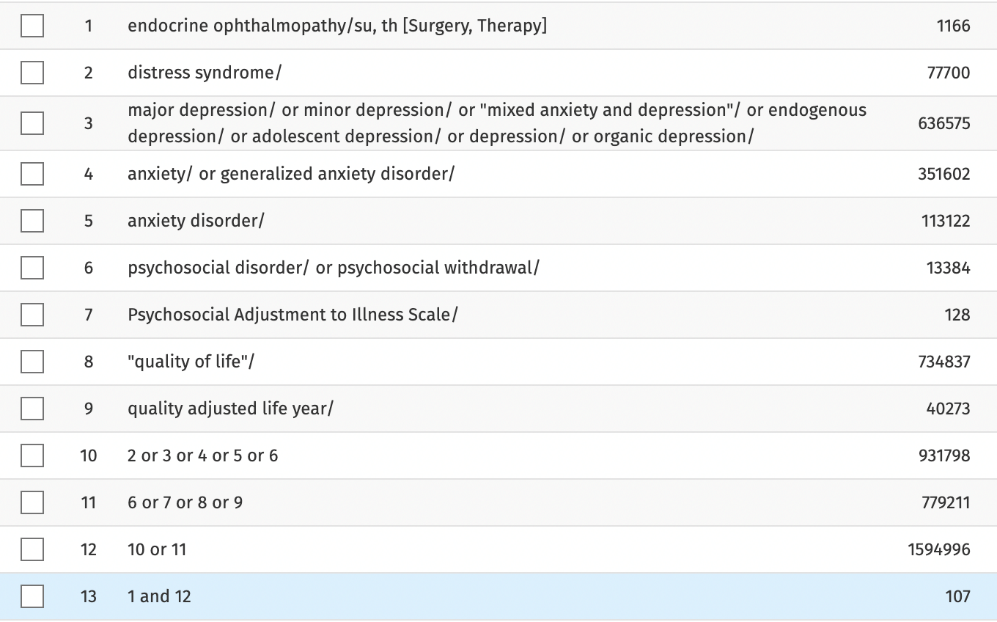


**CINAHL**

Date: 12/13/2022 updated 05/16/2025

**
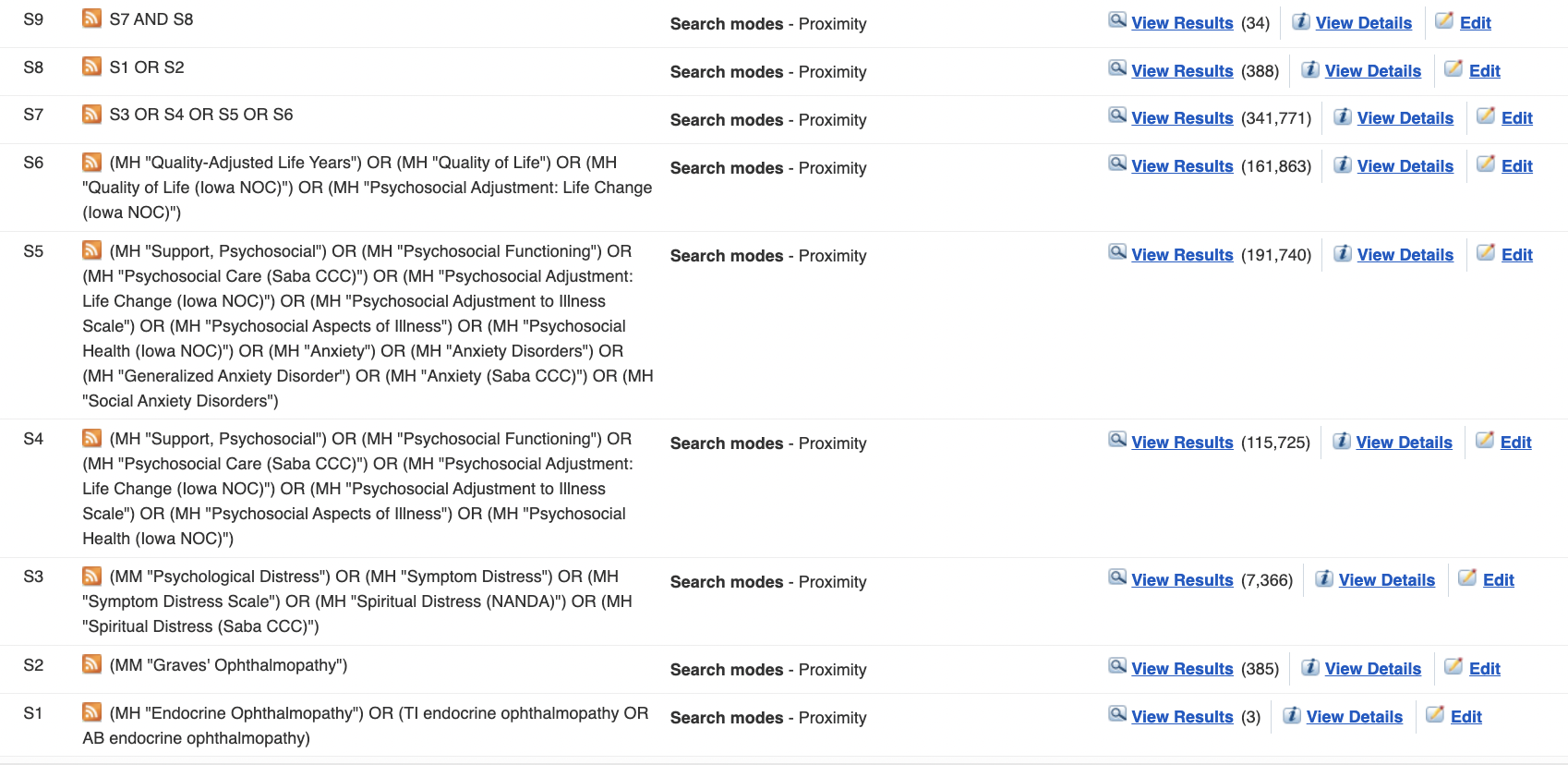
**

**Grey Literature**

1. **Clinical Trials.gov** (<https://clinicaltrials.gov/>)

Date: 08/29/2022

1. Search: (thyroid eye disease OR graves' eye disease OR graves' ophthalmopathy OR graves' orbitopathy OR endocrine ophthalmopathy) AND (psychological distress OR stress OR quality of life OR mental disorder)
2. 4 results
   1. <https://clinicaltrials.gov/ct2/show/NCT01893450?cond=%28thyroid+eye+disease+OR+graves%27+eye+disease+OR+graves%27+opthalmopathy+OR+graves%27+orbitopathy+OR+endocrine+opthalmopathy%29+AND+%28psychological+distress+OR+stress+OR+quality+of+life+OR+mental+disorder%29&rank=1>

🡪 Rejected: study terminated

- 1. <https://clinicaltrials.gov/ct2/show/NCT02393183?cond=%28thyroid+eye+disease+OR+graves%27+eye+disease+OR+graves%27+opthalmopathy+OR+graves%27+orbitopathy+OR+endocrine+opthalmopathy%29+AND+%28psychological+distress+OR+stress+OR+quality+of+life+OR+mental+disorder%29&rank=2> - complete in 2021

🡪 Rejected: lack of data – just beginning

- 1. <https://clinicaltrials.gov/ct2/show/NCT02203682>

à Rejected: published study included, which is available [here](https://www.ncbi.nlm.nih.gov/pmc/articles/PMC4499606/)

- 1. <https://clinicaltrials.gov/ct2/show/NCT03461211>

🡪 Rejected: no data available

1. **International Clinical Trials Registry Platform** (<https://www.who.int/clinical-trials-registry-platform>)

Date: 08/29/2022

1. Search: "graves eye disease" AND "quality of life"
2. 1 Result
3. <http://apps.who.int/trialsearch/Trial2.aspx?TrialID=NTR3796>

🡪 Rejected: recruiting, no results available

1. **Networked Digital Library of Theses & Dissertations (NDLTD)** (search.ndltd.org)

Date: 08/29/2022

- 1. Search: "graves eye disease" AND "quality of life"
  2. No results

1. **Pro-quest: Dissertations and Theses**

Date: 05/23/2022

1. Search: noft(graves eye disease) AND noft(quality of life)
2. 3 Results
   - 1. <https://www.lib.uwo.ca/cgi-bin/ezpauthn.cgi?url=http://search.proquest.com/docview/1827867541?accountid=15115>

🡪 Accepted

- - 1. <https://search-proquest-com.proxy1.lib.uwo.ca/docview/304650943/ABE2AF9C578A489APQ/1?accountid=15115>

🡪 Rejected: from 2000 and results not public (abstract only)

- - 1. <https://search-proquest-com.proxy1.lib.uwo.ca/docview/1873878690/ABE2AF9C578A489APQ/3?accountid=15115>

🡪 Rejected: no results available

1. **Conference Proceeding Searches**
   1. **The Association for Research in Vision and Ophthalmology** (ARVO) (<http://arvojournals.org/index.aspx>)
      1. Date: 08/10/2018
      2. Search: “meeting abstract” AND “thyroid eye disease” AND “quality of life”
      3. 1 Result

A. <https://iovs.arvojournals.org/article.aspx?articleid=2692950&resultClick=1>

🡪 Rejected: wrong outcome

1. AAO Meeting Archive (<https://secure.aao.org/aao/meeting-archive>)
   1. Date: 08/10/2018
   2. Search: “meeting abstract” AND “thyroid eye disease” AND “quality of life”
   3. No Results
2. Canadian Society of Ophthalmology (<http://www.cos-sco.ca/cpd/annual-meeting/>)
   1. Date: 08/10/2018
   2. Search: “meeting abstract” AND “thyroid eye disease” AND “quality of life”
   3. No Results

Appendix B. Screening Questions

1. Title/Abstract screening questions
2. Does the study involve thyroid eye disease/Graves eye disease/endocrine ophthalmology and QoL/stress/mental health?
3. Did the study take place after the year 2000?
4. Are at least 20 orbits assessed in the study?
5. Additional questions for full-text screening stage
6. Is the study available in English?
7. Are the study and its data accessible?

Appendix C. Kappa statistics calculations from screening stages (title and abstract).

Table C1. Kappa Statistics (Title Screening)

| **Review Authors** | **A.L.** | | | | |
| --- | --- | --- | --- | --- | --- |
| **B.Y.** |  | **Include** | **Exclude** | **Unsure** | **Total** |
|  | **Include** | 85 | 5 | 0 | **90** |
|  | **Exclude** | 52 | 118 | 50 | **220** |
|  | **Unsure** | 4 | 6 | 0 | **10** |
|  | **Total** | **141** | **129** | **50** | **320** |

$$Eq. \left( C.1 \right): Kappa=\frac{P\left( O \right)-P(E)}{1-P(E)}$$

$$P\left( O \right)=\frac{85+118+0}{320}$$

$P\left( O \right)=$0.634375

$$P\left( E \right)=\frac{\left( 90 x 141 \right)+\left( 220 x 129 \right)+(10 x 50)}{{320}^{2}}$$

$P\left( E \right)=$0.40595703

$$Given Eq. \left( C.1 \right):$$

$$Kappa=\frac{0.634375-0.40595703}{1-0.40595703}$$

$Kappa=$**0.38451422**

Table C2. Kappa Statistics (Abstract Screening)

| **Review Authors** | **A.L.** | | | | |
| --- | --- | --- | --- | --- | --- |
| **B.Y.** |  | **Include** | **Exclude** | **Unsure** | **Total** |
|  | **Include** | 64 | 13 | 0 | **77** |
|  | **Exclude** | 0 | 18 | 0 | **18** |
|  | **Unsure** | 0 | 7 | 0 | **7** |
|  | **Total** | **64** | **38** | **0** | **102** |

$$\mathrm{Using} Eq. \left( C.1 \right):$$

$$P\left( O \right)=\frac{64+18+0}{102}$$

$P\left( O \right)=$0.80392156

$$P\left( E \right)=\frac{\left( 77 x 64 \right)+\left( 18 x 38 \right)+(7 x 0)}{{102}^{2}}$$

$P\left( E \right)=$0.53940792

$$Kappa=\frac{P\left( O \right)-P(E)}{1-P(E)}$$

$$Kappa=\frac{0.80392156-0.53940792}{1-0.53940792}$$

$Kappa=$**0.574290484**

Table D. Quality checks using a modified Downs and Black checklist.

|  | | **Reporting** | **External Validity** | **Bias** | **Confounding** | **Power** |  |
| --- | --- | --- | --- | --- | --- | --- | --- |
| **Study** | **Year** | **Items 1-10** | **Items 11-13** | **Items 14-20** | **Items 21-26** | **Item 27*** | **Total (/27)** |
| Almeida et al. | 2024 | 8 | 2 | 6 | 5 | 1 | 22 |
| Bartalena et al. | 2012 | 9 | 3 | 7 | 5 | 0 | 24 |
| Bartalena et al. | 2017 | 7 | 3 | 7 | 5 | 1 | 23 |
| Cheng et al. | 2018 | 8 | 3 | 5 | 3.5 | 1 | 21.5 |
| Douglas et al. | 2020 | 9 | 3 | 7 | 6 | 1 | 27 |
| Fayers et al. | 2016 | 8 | 3 | 5 | 3 | 1 | 21 |
| Fichter et al. | 2013 | 8 | 3 | 5 | 1 | 1 | 18 |
| Jellema et al. * | 2014 | 9 | 3 | 5 | 3 | 1 | 21 |
| Jellema et al. * | 2017 | 9 | 3 | 5 | 3 | 1 | 21 |
| Kahaly et al. | 2005 | 10 | 3 | 5 | 4 | 1 | 24 |
| Kahaly et al. | 2005 | 9 | 3 | 5 | 4 | 1 | 22 |
| Kashkouli et al. | 2011 | 8 | 3 | 5 | 5 | 1 | 22 |
| Leon et al. | 2014 | 6 | 0 | 7 | 5 | 1 | 19 |
| Liang et al. | 2024 | 9 | 2 | 7 | 5 | 1 | 24 |
| Lin et al. | 2015 | 8 | 3 | 7 | 6 | 1 | 25 |
| Marcocci et al. | 2012 | 10 | 3 | 7 | 5 | 1 | 25 |
| Mourits et al. (EUGOGO) | 2009 | 9 | 3 | 5 | 3 | 1 | 21 |
| Potita et al. | 2024 | 7 | 1 | 5 | 4 | 1 | 18 |
| Prummel et al. | 2004 | 9 | 3 | 7 | 5 | 1 | 25 |
| Smith et al. | 2017 | 9 | 3 | 7 | 5 | 1 | 25 |
| Stoynova et al. | 2024 | 8 | 2 | 6 | 5 | 1 | 22 |
| Terwee et al. | 2011 | 6 | 3 | 5 | 3 | 1 | 18 |
| Zloto et al. | 2021 | 7 | 3 | 7 | 5 | 1 | 25 |

*Note: indicated studies are distinct studies involving different interventions from different years that happen to score similarly on quality check.

In our modified Downs and Blacks checklist, item 27 was only worth 1 point. The question used in place for item 27 was: 27. *Did the study have sufficient power to detect a clinically important effect where the probability value for a difference being due to chance is less than 5%?*

Appendix E. Tables summarizing QOL scores before and after treatments

Table E1. Mean visual function GO-QOL scores (VF GO-QOL) before and after various treatments.

| **Author** | **N** | **Participant Groups** | **Mean pre-treatment score (SD)** | **Mean post-treatment score (SD)** | **Follow-up** | ***p*** |
| --- | --- | --- | --- | --- | --- | --- |
| Almeida et al. | 21 | Inferomedial wall orbital decompression | 82.2 (17.2) | 90.5 (13.6) | 6 | 0.362 |
|  | 21 | medial plus lateral wall orbital decompression | 87.7 (14.4) | 82.8 (19.9) | 6 | 0.727 |
| Bartalena et al. | 53 | Methylprednisolone (low dosage: 2. 25g over 12 weeks) | 59 (30) | 61 | 2 | 0.11 |
|  |  |  | 59 (30) | 61 | 4 | 0.11 |
|  | 54 | Methylprednisolone (med dosage: 4.98 g over 12 weeks) | 55 (29) | 59.7 | 2 | 0.03 |
|  |  |  | 55 (29) | 65.1 | 4 | 0.03 |
|  | 52 | Methylprednisolone (high dosage: 7.47 g over 12 weeks) | 51 (29) | 58.3 | 2 | 0.01 |
|  |  |  | 51 (29) | 63.8 | 4 | 0.01 |
| Campi et al. | 100 | patients responsive to iV steroid therapy (ivGC) | 74.2 (30) | 84 (28) | 6 | 0.05 |
|  |  | patients non-responsive to ivGC | 69.3 (27) | 58.1 (18.5) | 6 | 0.05 |
|  |  | patients responsive to squint surgery | 47.4 (27.3) | 76.6 (17.6) | 3 | 0.01 |
|  |  | patients non-responsive to squint surgery | 52.1 (24.8) | 71.3 (20.6) | 3 | 0.01 |
| Cheng et al. | 43 | Decompression surgery (fat) | 54.5 (30.5) | 66.3 (27.6) | 6 | 0.01 |
| Fayers et al. | 29 | Decompression surgery | 55.7 (27.1) | 54.4 (26.1) | - | 0.94 |
| Fayers et al. | 15 | Strabismus surgery | 41.3 (21.3) | 70.2 (18.9) | - | 0.0048 |
| Fayers et al. | 26 | Eyelid procedure (palpebral aperture narrowing) | 67.3 (26.6) | 71.7 (26.6) | - | 0.1244 |
| Fichter et al. | 18 | Decompression surgery | 34.1 (31.4) | 48.5 (35.9) | 3 | 0.016 |
|  |  |  | 34.1 (31.4) | 69 (38.3) | 6 | 0.016 |
| Hoppe et al. | 100 | Methylprednisolone | 66.5 (24.5) | 68.7 (18.9) | 3 | 0.967 |
|  |  | Methylprednisolone | 66.5 (24.5) | 69.6 (24.9) | 6 | 0.01 |
|  |  | Methylprednisolone | 66.5 (24.5) | 72.2 (25.8) | 9 | 0.04 |
| Jellema et al. | 26 | Strabismus surgery | 46 (26) | 66 (31) | 2 | 0.009 |
| Kashkuoli et al. | 61 | Glucocorticoids | 62.3 | 82.4 | 6 | - |
|  | 61 | Decompression surgery | 43.5 | 78.1 | 6 | 0.06 |
| Liang et al. | 82 | Patients with dysthyroid optic neuropathy | 33.18 (24.54) | - | - | 0.001 |
|  | 82 | Patients with non-dysthyroid optic neuropathy | 81.26 (17.39) | - | - | 0.001 |
| Lin et al. | 13 | Doxycycline | 61.99 | 65.02 | 3 | 0.21 |
|  | 13 | Doxycycline | 61.99 | 71.64 | 6 | 0.21 |
| Marcocci et al. | 152 | Selenium | 80.1 (17.1) | 88.83 (17.7) | 6 | 0.001 |
|  | 152 | Selenium | 80.1 (17.1) | 91.1 (15.3) | 12 | 0.004 |
|  | 152 | Placebo | 80.1 (17.1) | 77.1 (14.6) | 6 | 0.001 |
|  | 152 | Placebo | 80.1 (17.1) | 78.4 (18.7) | 12 | 0.004 |
| Potita et al. | 13 | Selenium | 68.5 (30.1) | 77.6 (28.8) | 6 | 0.06 |
|  | 13 | Placebo | 82.9 (17.7) | 76.7 (22.7) | 6 | 0.3 |
| Prummel et al. | 88 | Radiotherapy | 60.4 (23.2) | 68.6 (15.8) | 12 | - |
|  | 88 | Placebo | 62.5 (23.1) | 73 (16.8) | 12 | - |
| Smith et al. | 87 | Teprotumumab | 34.5 (7.3) | 52.2 (2.4) | 2 | 0.001 |
| Stoynova et al. | 221 | Graves’ disease | 59.1 (28.3) | - | - | 0.448 |
|  | 221 | Hyperthyroid | 57.6 (29.5) | - | - | 0.448 |
| Smith et al. | 87 | Placebo | 34.5 (7.3) | 41.3 (2.3) | 2 | 0.001 |
| Terwee et al. | 23 | Radiotherapy | 37 (20.7) | 45.1 (26.9) | 3 | 0.05 |
|  | 10 | Decompression surgery (for sight loss) | 27.1 (22.4) | 47.4 (28.3) | 6 | - |
|  | 38 | Decompression surgery (for exophthalmos) | 64.8 (23.9) | 68 (22.8) | 6 | - |
|  | 31 | Eye muscle surgery | 50.5 (23.3) | 53.3 (28.9) | 6 | - |
|  | 43 | Eye lid surgery (lengthening) | 66.7 (26.9) | 70.4 (2.1) | 6 | - |
|  | 19 | Eye lid surgery (blepharoplasty) | 64.7 (29.7) | 64.9 (27.4) | 6 | - |

Table E2. Mean visual appearance GO-QOL scores (VA GO-QOL) before and after various treatments.

| **Author** | **N** | **Participant Groups** | **Mean pre-treatment score (SD)** | **Mean post-treatment score (SD)** | **Follow-up** | ***p*** |
| --- | --- | --- | --- | --- | --- | --- |
| Almeida et al. | 21 | Inferomedial wall orbital decompression | 67.4 (17.6) | 73.5 (17.1) | 6 | 0.675 |
|  | 21 | medial plus lateral wall orbital decompression | 60.9 (!5.7) | 81.3 (12.9) | 6 | 0.006 |
| Campi et al. | 100 | patients responsive to IV steroid therapy (IVGC) | 84.1 (27) | 96.4 (20) | 6 | 0.01 |
| Campi et al. | 100 | patients non-responsive to IVGC | 113.9 (17) | 101.1 (25) | 6 | 0.01 |
| Campi et al. | 100 | patients responsive to squint surgery | 61.5 (24.8) | 81.9 (19.4) | 3 | 0.01 |
| Campi et al. | 100 | patients non-responsive to squint surgery | 63.9 (24.2) | 76.3 (35.7) | 3 | 0.01 |
| Cheng et al. | 43 | Decompression (fat) | 39.8 (26.2) | 56.3 (21.8) | 6 | 0.001 |
| Fayers et al. | 29 | Decompression surgery | 35.6 (25.7) | 49.9 (28.7) | - | 0.038 |
| Fayers et al. | 15 | Strabismus surgery | 50.8 (26.3) | 64.6 (21.6) | - | 0.016 |
| Fayers et al. | 26 | Eyelid procedure (palpebral aperture narrowing) | 51.4 (25.9) | 69.8 (25.7) | - | 0.0003 |
| Hoppe et al. | 100 | Methylprednisolone | 66.5 (24.5) | 68.7 (18.9) | 3 | 0 |
| Hoppe et al. | 100 | Methylprednisolone | 66.5 (24.5) | 69.6 (24.9) | 6 | 0.01 |
| Hoppe et al. | 100 | Methylprednisolone | 66.5 (24.5) | 72.2 (25.8) | 9 | 0.005 |
| Jellema et al. | 26 | Strabismus surgery | 61 (26) | 71 (22) | 2 | 0.005 |
| Liang et al. | 82 | Patients with dysthyroid optic neuropathy | 60.08 (24.82) | - | - | 0.001 |
|  | 82 | Patients with non-dysthyroid optic neuropathy | 76.14 (27.56) | - | - | 0.001 |
| Lin et al. | 13 | Doxycycline | 46.17 | 64.45 | 3 | 0.0008 |
|  | 13 | Doxycycline | 46.17 | 67.76 | 6 | 0.0008 |
| Marcocci et al. | 152 | Selenium | 74 (19.8) | 84.6 (10.9) | 6 | 0.001 |
|  | 152 | Selenium | 74 (19.8) | 86.6 (11.8) | 12 | 0.001 |
|  | 152 | Placebo | 79.5 (18.1) | 76.9 (11.7) | 6 | 0.001 |
|  | 152 | Placebo | 79.5 (18.1) | 77.9 (17.1) | 12 | 0.001 |
| Potita et al. | 13 | Selenium | 43.7 (31.9) | 55.6 (27.5) | 6 | 0.04 |
|  | 13 | Placebo | 64.1 (28) | 65.6 (27.9) | 6 | 0.81 |
| Prummel et al. | 88 | Radiotherapy | 53.2 (27.5) | 59.9 (17.2) | 12 | - |
|  | 88 | Placebo | 53 (25.9) | 58.5 (16.6) | 12 | - |
| Stoynova et al. | 221 | Graves’ disease | 56.5 (24.8) | - | - | 0.56 |
|  | 221 | Hyperthyroid | 62.1 (26.1) | - | - | 0.56 |
| Terwee et al. | 23 | Radiotherapy | 72 (18.6) | 73.6 (22.7) | 3 | - |
|  | 10 | Decompression surgery (for sight loss) | 51 (19.8) | 55 (15) | 6 | 0.001 |
|  | 38 | Decompression surgery (for exophthalmos) | 44.7 (24.4) | 55.8 (26.7) | 6 | - |
|  | 31 | Eye muscle surgery | 65.1 (20.7) | 67.7 (24.4) | 6 | - |
|  | 43 | Eye lid surgery (lengthening) | 63.4 (22.9) | 67.6 (21.9) | 6 | - |
|  | 19 | Eye lid surgery (blepharoplasty) | 58.6 (29.6) | 68.8 (24.1) | 6 | 0.02 |

Table E3. Mean overall GO-QOL scores before and after various treatments.

| **Author** | **N** | **Participant Groups** | **Mean pre-treatment score (SD)** | **Mean post-treatment score (SD)** | **Follow-up** | ***p*** |
| --- | --- | --- | --- | --- | --- | --- |
| Hoppe et al. | 100 | Methylprednisolone | 65.1 (16.7) | 68.7 (18.9) | 3 | 0.012 |
| Hoppe et al. | 100 | Methylprednisolone | 65.1 (16.7) | 70.4 (22.2) | 6 | 0.028 |
| Hoppe et al. | 100 | Methylprednisolone | 65.1 (16.7) | 71.8 (22.4) | 9 | 0.004 |
| Zloto et al. | 10 | IVGC (high dose, 1g daily for 3 days) | 46.11 | 65.64 | 29 | 0.07 |
| Zloto et al. | 23 | IVGC (EUGOGO) | 67.08 | 90 | 27.76 | 0.04 |
| Zloto et al. | 6 | Immunosuppressant | 52.33 | 70.24 | 30.23 | 0.352 |
| Zloto et al. | 16 | Decompression | 70 | 75 | 32.83 | 0.021 |
| Zloto et al. | 3 | Strabismus sugery | 57.33 | 93 | 31.73 | 0.042 |
| Zloto et al. | 18 | Eyelid surgery | 69.91 | 75 | 18.27 | 0.213 |
| Zloto et al. | 38 | Average for all medical treatment | 62.18 | 80.71 | 30.86 | 0.049 |
| Zloto et al. | 28 | Average for all surgical treatments | 59.5 | 77.3 | 30.86 | 0.047 |
| Zloto et al. | 90 | No medical or surgical treatment | 69.91 | 75.65 | 18.27 | 0.497 |
